# Identification and Quantitation of ^14^C-Labeled Catechol Metabolites in Rat Plasma After Intranasal Instillation of Smoldering Eucalyptus Wood Smoke Extract

**DOI:** 10.3390/mps8060147

**Published:** 2025-12-04

**Authors:** David Baliu-Rodriguez, Dorothy J. You, Michael A. Malfatti, Esther A. Ubick, Yong Ho Kim, Bruce A. Buchholz

**Affiliations:** 1 Biosciences and Biotechnology Division, Lawrence Livermore National Laboratory, Livermore, CA 94550, USAmalfatti1@llnl.gov (M.A.M.);; 2 Risk Assessment Support Division, Office of Mission Critical Operations, Office of Chemical Safety and Pollution Prevention, U.S. Environmental Protection Agency, Research Triangle Park, NC 27711, USA; kim.yongho@epa.gov; 3 Center for Accelerator Mass Spectrometry, Lawrence Livermore National Laboratory, Livermore, CA 94550, USA

**Keywords:** catechol, wildfire, wood smoke extract, liquid chromatography-mass spectrometry, LC-MS, accelerator mass spectrometry, AMS, parallel accelerator and molecular mass spectrometry, PAMMS, carbon-14

## Abstract

The increasing frequency, duration, and intensity of wildfires over the past decade have raised significant concerns about widespread exposure to wildfire smoke. Inhalation of wildfire smoke poses a substantial risk to human health, with epidemiological studies linking exposure to cardiovascular, respiratory, and neurological dysfunction. Wildfire smoke contains hundreds of chemical compounds across diverse classes, with concentrations varying by fuel type and combustion conditions. Phenolic compounds are prominent constituents of wood smoke, and catechol is especially abundant under smoldering conditions that produce dense smoke. In this study, ^14^C-labeled catechol was spiked into smoldering eucalyptus wood smoke extract (WSE) and administered to rats via intranasal instillation. Plasma was collected at 5 min and 2 h post-exposure. Samples were analyzed using parallel accelerator and molecular mass spectrometry (PAMMS). Major catechol-derived metabolites identified included benzene oxide, catechol-cysteine conjugate, and catechol-glutamine conjugate; the parent compound was not detected. These results indicate that inhaled catechol in wood smoke is quickly metabolized upon entry into circulation. PAMMS enabled both identification and relative quantification of circulating catechol metabolites, demonstrating feasibility for biomarker discovery and exposure assessment.

## 1. Introduction

Wildfire activity has intensified in frequency, duration, and geographic reach, exposing large populations to complex mixtures of combustion products [1,2]. Inhalation of wildfire smoke is associated with increased cardiopulmonary morbidity and all-cause mortality, and emerging evidence implicates neurological effects as well [3,4]. Yet mechanistic understanding of which chemical constituents reach the circulation, how they are metabolized, and which species are most informative as exposure biomarkers remains limited, largely because smoke is chemically heterogeneous and dynamic.

Phenolic compounds derived from lignin are major components of wood smoke, particularly under smoldering conditions that generate dense, particle-rich plumes [5,6]. Among these, catechol is frequently abundant [7]. Mice exposed to smoke from ^3^H-catechol spiked cigarettes showed rapid absorption and clearance of ^3^H in blood, and >90% of the activity was excreted in urine within 2 h [8]. Once inhaled, catechol is rapidly oxidized to electrophilic o-quinones and undergoes conjugation (e.g., with amino acids and glutathione), yielding transient intermediates and downstream mercapturic acids [9,10,11]. Capturing these short-lived species in vivo and linking them to an exposure source requires analytical strategies that provide both molecular specificity and quantitative source tracing.

To address these challenges, we apply Parallel Accelerator and Molecular Mass Spectrometry (PAMMS) to blood/plasma for the direct, chromatographic tracking of ^14^C-labeled molecules and their circulating metabolites. PAMMS couples liquid chromatography to a moving wire, liquid-sample accelerator mass spectrometry (AMS) detector for ultra-sensitive, compound-agnostic quantification of radiocarbon in real time [12,13], in parallel with high-resolution (HR) molecular MS for structural information [14]. In practice, LC effluent is split: one path enters the moving wire interface and AMS quantifies the total ^14^C signal as peaks, while the other path is interrogated by HRMS to acquire full-scan and/or tandem MS data. The chromatographic co-elution of ^14^C peaks with molecular features enables unambiguous linkage between radiocarbon-bearing species and their molecular ions, even when the parent compound is fully transformed. AMS is the most sensitive analytical technique to measure ^14^C and well suited to this application [15,16,17,18,19,20,21].

This configuration is novel in the blood/plasma matrix because it (i) achieves sub-attomole sensitivity for ^14^C, enabling detection of metabolites following environmentally relevant or microtracer doses that are typically far below the limits required for conventional LC-MS/MS toxicokinetic studies; (ii) requires no prior knowledge of metabolite structures, since all ^14^C-bearing species are visible to AMS independent of ionization efficiency or fragmentation behavior; (iii) preserves temporal resolution suitable for early pharmacokinetic sampling; and (iv) minimizes sample handling, avoiding bulk fraction collection or off-line radiometric assays.

In conventional LC-MS/MS workflows, quantitative analysis typically relies on authentic standards and achieves limits of quantification in the µmol-pmol range, depending on ionization efficiency and instrumentation. By contrast, the AMS component of PAMMS can routinely detect <1 amol of ^14^C per sample injection. This sensitivity allows toxicokinetic profiling at doses orders of magnitude lower than standard animal studies, including environmentally relevant exposures that would fall below the detection limits of even the most sensitive triple quadrupole or Orbitrap instruments. As a result, PAMMS simultaneously delivers source tracing (via ^14^C) and structural assignment (via HRMS) in a single LC run from small plasma volumes, making it possible to track transient, low-abundance metabolites without requiring a priori knowledge or high dosing.

Beyond catechol, the workflow is broadly applicable to ADME/PK studies of any ^14^C-labeled xenobiotic and across different exposure routes (inhalation, oral, dermal). AMS peak areas can be integrated to derive C_max_, T_max_, AUC, and clearance-relevant metrics for the total radiolabel and, when resolved chromatographically, for individual ^14^C-carrying metabolites [22]. In parallel, orbitrap data inform putative identification (accurate mass, isotope pattern, formula enumeration, MS/MS) and can be escalated to targeted confirmation as needed. Thus, PAMMS enables end-to-end tracing from exposure to circulating molecular species, supporting biomarker discovery, toxicokinetic modeling, and translational risk assessment.

Here, we demonstrate this capability by administering ^14^C-catechol via intranasal instillation to rats and applying PAMMS to track circulating, radiolabeled species in plasma. We provide a method to facilitate adoption of PAMMS for blood-based metabolite tracing in smoke-relevant and broader ADME/PK applications.

## 2. Materials and Methods

### 2.1. Chemicals

^14^C-Catechol was purchased from American Radiolabeled Chemicals (St. Louis, MO, USA) and diluted with unlabeled catechol (Thermo Fisher, Waltham, MA, USA) to a working specific activity of 30.690 µCi/mmol and spiked into wood smoke extract (WSE) at a concentration of 150 µg/mL to deliver a catechol dose of 50 µg/kg in 100 µL to a 0.30 kg rat. Catechol is often the most plentiful phenolic compound in wildfire smoke and the ^14^C-catechol spike added to WSE served as chemical class tracer for phenolic compounds. The spike concentration falls in the middle of the range of phenolics reported in field measurements (0.5–26%) in wildfire smoke [23,24,25,26].

### 2.2. Smoldering Eucalyptus Wood Smoke Extracts

WSE were generously provided by the US EPA. Wood smoke extracts were generated by burning eucalyptus in a customized furnace and collected during the smoldering phase of the burning as described in Kim et al. [27,28]. WSE was provided in acetone and solvent exchange was performed under nitrogen atmosphere for resuspension in 0.9% NaCl (Thermo Fisher) at 1 mg/mL. Resuspended WSE was aliquoted and frozen at −20 °C before use.

### 2.3. Animals

All animal experiments were conducted at the Lawrence Livermore National Laboratory (LLNL) AAALAC accredited animal care facility. The protocol for the animal experiments was reviewed and approved (protocol 23-06-062) by the LLNL Institutional Animal Care and Use Committee (IACUC) in June 2023 prior to the study and reviewed annually by the IACUC. Male Sprague Dawley rats weighing 250–300 g with a surgically implanted jugular vein catheter were obtained from Envigo (Indianapolis, IN). Rats were housed individually in polystyrene cages containing hardwood bedding and kept on a 12 h light/dark cycle in a ventilated room maintained at 24 °C. Food and water were provided ad libitum.

### 2.4. Animal Exposures

Male Sprague Dawley rats (n = 5) were anesthetized with 4–5% isoflurane in 100% oxygen and intranasally instilled with ^14^C-catechol at a dose of 50 µg/kg, suspended in wood smoke extract (WSE), at a concentration of 1 mg/mL. The doses are based on documented air quality measurements during wildfire events such as the 2018 Camp Fire in California where PM2.5 was measured >400, 220, and 195 µg/m^3^ at distances ~20 km, 140 km and 240 km, respectively, from the fire [29]. An adult 70 kg human has a ventilation rate of 6 L/min at rest and inhales 8640 L of air in 24 h [30]. When the PM concentration is 200 µg/m^3^, a value common during wildfire events, the mass of PM inhaled in 24 h equals 1.73 mg. The dose to a 70 kg human is then 25 µg/kg. Allometric scaling is utilized when comparing doses to different species to account for the faster metabolism rates of smaller animals and results in higher dose/mass in the smaller animal. The scaling factor from human to rat is 6.2 [31], resulting in a rat dose of 155 µg/kg. A 0.3 kg mouse then receives 24 h dose of 46.5 µg of PM. Deposition fraction by both inhalation and intranasal installation is less than 100% but was not quantified. The total dose (100 µg WSE + 15 µg catechol) is equivalent using allometric scaling [31] to 2.5 days of human exposure to 200 µg/m^3^ particulate matter (PM), a level common during wildfire events. Animals were randomly assigned to vehicle only control or WSE dose. Control animals (n = 5) received an equivalent volume (~100 µL) of vehicle solution (0.9% NaCl) under identical anesthesia conditions. Blood samples (~0.3 mL) were collected from the jugular vein at baseline (pre-dose) at 5 min and 2 h post-dose, and transferred to lithium heparin-coated Microtainer^®^ tubes (Becton Dickinson, Franklin Lakes, NJ, USA). Samples were immediately refrigerated at 4 °C. Within one hour of collection, plasma was separated by centrifugation at 8000× *g* for 2 min, following the manufacturer’s recommendations. Plasma samples were stored at −20 °C until analysis.

A power calculation was used to determine that a sample size of 10 would provide sufficient information for proper assessment of the endpoints as well as an assessment of animal-to-animal variation based on prior studies [28,32]. The plasma from five randomly chosen animals was chosen for PAMMS analysis as a representative sample. A random number generator was used for randomization. Confounders were not controlled. No criteria were set for animal inclusion or exclusion, and there were no exclusions. Blinding was not used for downstream sample analysis.

### 2.5. Plasma Preparation

Analytes were extracted and purified from plasma using solid-phase extraction (SPE) with C18 cartridges containing 130 mg sorbent beds (Waters; Milford, MA, USA). The SPE method was adapted from a previously developed method [33]. C18-based SPE has been used for the extraction of catechol from environmental matrices [34], as well as for the enrichment of catechol metabolites [35] and structurally related phenolic compounds (e.g., benzene [36] and chlorinated phenols [37]) from biological samples, supporting its suitability for this application. Cartridges were pre-washed with 1 mL of 90:10 methanol:water (*v*/*v*) containing 0.1% formic acid, followed by conditioning with 1 mL of water containing 0.1% formic acid. Forty microliters of plasma were diluted in 1 mL of water containing 0.1% formic acid and loaded onto the cartridge. After sample loading, the cartridge was washed with 1 mL of water containing 0.1% formic acid. Analytes were eluted with 1.8 mL of 90:10 methanol:water (*v*/*v*) containing 0.1% formic acid. Eluates were dried under vacuum and stored at −20 °C. Prior to analysis, samples were reconstituted in 100 µL of 95:5 water:acetonitrile (*v*/*v*) containing 0.1% formic acid.

### 2.6. Parallel Accelerator and Molecular Mass Spectrometry

LC-MS analyses were conducted using a Vanquish UHPLC coupled to an Orbitrap Eclipse mass spectrometer (Thermo Fisher). The LC was equipped with an autosampler, binary pump, and column compartment. Reverse-phase (RP) separation was achieved using a Thermo Accucore RP-MS column (2.1 × 100 mm, 2.6 µm particle size). Gradient elution was employed with mobile phase A consisting of 95:5 water:acetonitrile (*v*/*v*) with 10 mM ammonium formate and 0.125% formic acid, and mobile phase B consisting of 5:95 water:acetonitrile (*v*/*v*) with 0.125% formic acid. The gradient was as follows: 0–1 min, 0% B; 1–5 min, linear increase to 40% B; 5–6 min, linear increase to 100% B; 6–7.5 min, hold at 100% B; 7.5–13 min, re-equilibrate at 0% B. The flow rate was set to 0.250 mL/min from 0 to 7.5 min and 0.400 mL/min from 7.5 to 13 min. The column temperature was maintained at 40 °C and the injection volume was 5 µL. A flow splitter was used to divert half of the eluent (0.125 or 0.200 mL/min) to the Orbitrap Eclipse for molecular mass spectrometry, and the remainder to the moving wire interface for accelerator mass spectrometry.

AMS analysis was performed using LLNL’s custom-built moving wire interface [12,38]. Briefly, the LC eluent was continuously deposited onto a moving nickel wire, which then passed through a drying oven maintained at 120 °C to remove solvent and other volatile compounds. The dried sample was combusted in an 800 °C oven, converting carbon-containing species to CO_2_. The CO_2_ was transferred via capillary to the AMS source. A 250 kV AMS (National Electrostatics Corporation; Middleton, WI, USA) was used to detect and quantify both stable and radiocarbon isotopes. Data were analyzed using in-house software.

Molecular MS was performed using heated electrospray ionization (HESI) in negative ion mode. The spray voltage was set to 2500 V, with the RF lens at 50%. Sheath gas, auxiliary gas, and sweep gas were set to 35, 7, and 0 arbitrary units, respectively. The ion transfer tube temperature was 300 °C, and the vaporizer temperature was 275 °C. Full-scan acquisition was conducted at a resolution of 120,000 *m*/*z* with a mass range of 80–800 *m*/*z*. Fluoranthene was used as an internal calibrant. Data were collected from 0–7.5 min and analyzed using FreeStyle software version 1.8 (Thermo Fisher).

MS/MS experiments were performed using both collision-induced dissociation (CID) and higher-energy collisional dissociation (HCD). For CID, the collision energy was fixed at 30%, while for HCD the collision energy was stepped at 15%, 30%, and 45%. In both fragmentation modes, precursor ions were isolated in the quadrupole with a 1.6 *m*/*z* isolation window, and product ions were detected in the orbitrap mass analyzer at a resolution of 60,000. Mass ranges were 40–229 *m*/*z*.

Chromatograms and mass spectra shown in figures are qualitatively representative of results from all ten samples, though relative peaks areas differed.

## 3. Results and Discussion

**Figure 1:**
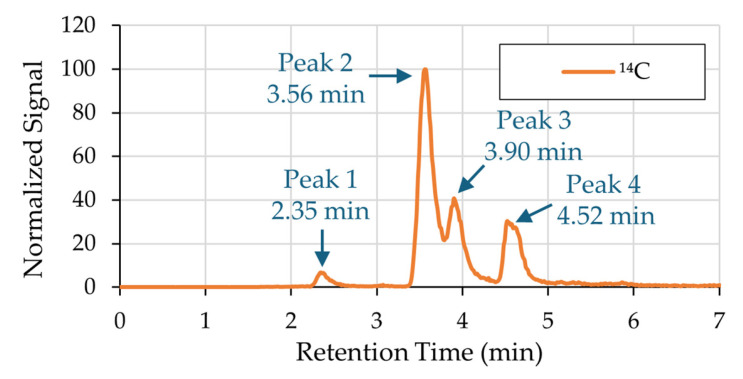
A representative ^14^C chromatogram obtained by PAMMS analysis of rat plasma after ^14^C-labeled catechol administration. All ten analyzed samples had a consistent chromatographic profile characterized by four distinct radiocarbon peaks. The peaks were numbered 1 through 4 by order of elution.

Plasma was collected from five rats 5 min and 2 h after intranasal instillation of ^14^C-labeled catechol (10 total samples). The plasma was purified with solid-phase extraction and analyzed for ^14^C quantitation and molecular identification using PAMMS. Figure 1 displays a representative ^14^C chromatogram obtained by PAMMS, and Figure S1 includes the ^14^C chromatogram overlaid on the total ion chromatogram (TIC). All ten chromatograms generated across biological replicates exhibited a consistent profile, characterized by four distinct radiocarbon peaks, labeled Peak 1 through Peak 4, eluting at reproducible retention times. Mass spectra from the Orbitrap MS were investigated to find ions with matching peak shapes and retention times. Neither catechol nor ^14^C above background levels were detected in control plasma.

### 3.1. ^14^C-Labeled Compound Identification

**Figure 2:**
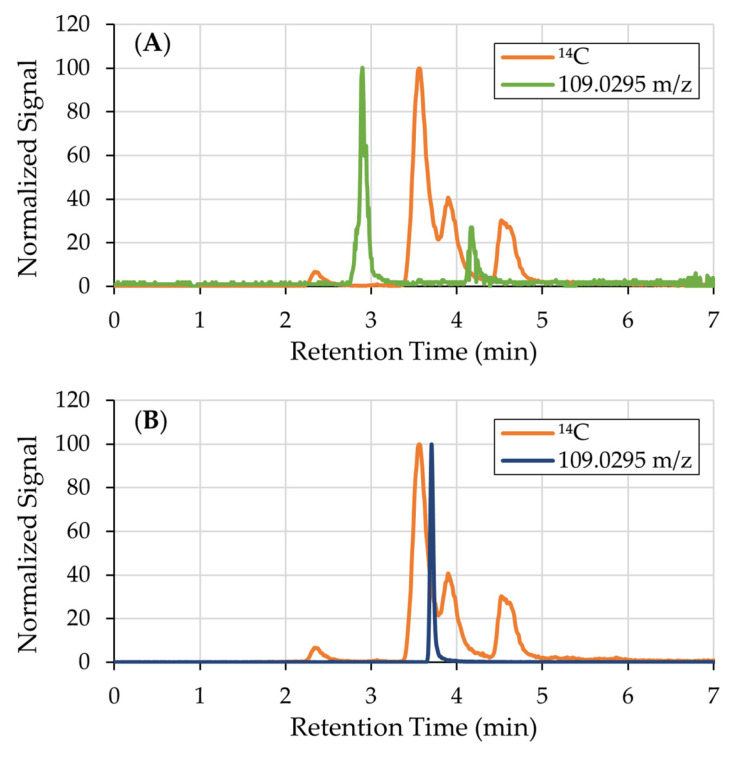
(**A**) ^14^C chromatogram overlaid with the EIC of catechol, 109.0295 *m*/*z*. Though there are ions with 109.0295 *m*/*z*, they are not radiolabeled and therefore not derived from catechol. (**B**) EIC of catechol (RT = 3.70 min) overlaid on the ^14^C chromatogram.

Although ions with the [M-H]^−^ of catechol, 109.0295 *m*/*z*, were detected, they were not radiolabeled and therefore not derived from ^14^C-catechol (Figure 2A). Additionally, the retention time of these ions did not match that of the spiked catechol standard (3.70 min, Figure 2B), suggesting that they may represent structural isomers such as hydroquinone or resorcinol. Alternatively, these ions may have arisen from in-source fragmentation during the ionization process.

Assignment of Peak 1 was initiated by aligning the ^14^C AMS peak at 2.35 min with orbitrap full-scan data. The only feature that co-eluted and reproduced the ^14^C peak shape was 218.1034 *m*/*z* (RT = 2.34 min). Its isotopic pattern (no elevated A+2 signal) indicated a C/H/N/O composition. FreeStyle software version 1.8 was then used for elemental-formula suggestion at 218.1034 *m*/*z*, returning several candidates ranked by mass accuracy, isotope fit, and heuristic constraints (Table 1). The top candidate was the [M-H]^−^ ion of C_9_H_17_O_5_N, with Δ*m*/*z* = 0.0000, a rings plus double bonds equivalents (RDB) of 2.0 consistent with a moderately unsaturated, highly polar species expected to elute early with reverse-phase chromatography, and the highest combined score (84.52) among candidates. Alternative formulas were less plausible: C_8_H_11_N_8_ (RDB = 7.5) is nitrogen-rich and highly unsaturated for this biological context; C_10_H_21_NS_2_ (RDB = 1.0) contains two sulfurs and would show an A+2 isotope enhancement that was absent; and H_15_O_2_N_10_S (RDB = −1.5) is chemically unrealistic for a closed-shell small molecule.

MS/MS data (Figure S2) corroborated the C_9_H_17_O_5_N assignment. HCD of the 218.1034 m/z precursor produces a dominant product ion at 146.0822 *m*/*z*, which was assigned the formula C_6_H_12_O_3_N^−^ (Δ*m*/*z* = 0.0010). The loss of C_3_H_4_O_2_ corresponds to cleavage of the catechol and retention of the glutamine backbone. Secondary glutamine-derived product ions appear at 116.0716 m/z (C_5_H_10_O_2_N^−^, Δ*m*/*z* = 0.0010) and 88.0403 m/z (C_3_H_6_O_2_N^−^, Δ*m*/*z* = 0.0010), generated by successive neutral losses typical of amino-acid side-chain rearrangements. Complementary catechol-derived ions are observed at 99.0450 *m*/*z* (C_5_H_7_O_2_^−^, Δ*m*/*z* = 0.0009) and 71.0501 m/z (C_4_H_7_O^−^, Δ*m*/*z* = 0.0010), while 71.0137 *m*/*z* (C_3_H_3_O_2_^−^, Δ*m*/*z* = 0.0010) represents a low-*m*/*z* ring-fragment of the phenolic moiety. All fragment masses deviate from their theoretical values by ≤ 0.0010 *m*/*z*, and none contain sulfur or multiple nitrogens, features expected for the lower-ranked elemental formulas but absent here. The presence of paired glutamine- and catechol-specific fragments provides mechanistic evidence that the precursor consists of a catechol group covalently linked to glutamine. Thus, the MS/MS spectrum reinforces the chromatographic, isotopic, and elemental composition evidence, confirming Peak 1 as the [M-H]^−^ ion of a catechol-glutamine conjugate (C_9_H_17_O_5_N).

**Table 1:** Elemental formula predictions for the ion at 218.1034 *m*/*z* generated by Thermo Scientific FreeStyle software. Candidate formulas are ranked by mass accuracy (*m*/*z* delta), rings plus double bonds equivalents (RDB), and a combined score that integrates isotopic fit, heuristic rules, and elemental plausibility.

Formula	[M-H]^−^	*m*/*z* Delta	RDB	Combined Score
C_9_H_17_O_5_N	218.1034	0.0000	2.0	84.52
C_8_H_11_N_8_	218.1034	0.0000	7.5	83.83
C_10_H_21_NS_2_	218.1043	−0.0009	1.0	82.51
H_15_O_2_N_10_S	218.1027	0.0007	−1.5	74.93

For the second ^14^C peak, EICs were generated around the candidate [M-H]^−^ ions and overlaid with the AMS trace. The only feature that coeluted (RT = 3.56 min) with the ^14^C peak and reproduced its shape was 93.0346 *m*/*z*, which FreeStyle ranked highest among elemental formula candidates (Table 2). The top hit, C_6_H_6_O ([M-H]^−^ = 93.0335 *m*/*z*), showed a small *m*/*z* delta and the highest combined score (89.71), as well as an RDB of 4.0, consistent with one ring and three double bonds, such as in catechol, or two rings and two double bonds, as expected for an epoxide metabolite of catechol. Competing assignments were less plausible: C_4_H_4_N_3_ (RDB = 4.5) implies an unusually nitrogen-dense structure for this biological context; H_7_N_4_P and C_2_H_9_ONP require phosphorus and multiple nitrogens that are not supported by the retention time. Targeted CID and HCD (Figure S3) did not yield product ions above the method’s acquisition limit (40 *m*/*z*), which is expected for a small, resonance-stabilized anion such as C_6_H_5_O^−^. Dissociation channels either produce neutral losses or fragments that fall below the first-mass setting, or require higher energies that deplete precursor without generating abundant, informative products. Although alternative approaches such as ultraviolet photodissociation (UVPD), electron capture/transfer dissociation (ECD/ETD), or infrared multiphoton dissociation (IRMPD) can provide richer fragmentation for larger or multiply charged species, they would not overcome the low-mass cutoff limitation, which is instrument defined and independent of fragmentation mechanism. In other words, even if additional dissociation pathways were introduced, the resulting fragments for this low-*m*/*z* ion would still fall below the detectable range, and therefore would not yield structurally informative spectra. Thus, the lack of MS/MS signal reflects the intrinsic mass range and stability of the ion, rather than a limitation of the dissociation chemistry.

The absence of adducts or co-eluting in-source fragments at 3.56 min, together with agreement in retention time and peak shape with the ^14^C signal and the FreeStyle score metrics, supports a tentative assignment of Peak 2 as benzene oxide (C_6_H_6_O), a well-recognized intermediate formed during oxidative metabolism of aromatic substrates. This assignment aligns with the biological context of catechol-derived oxidation chemistry in vivo.

The ion 212.0021 *m*/*z* (RT = 3.88 min) co-eluted with the third ^14^C peak and reproduced its shape. FreeStyle ranked C_8_H_7_O_4_NS as the best candidate for molecular formula (Table 3), with Δ*m*/*z* = −0.0002, the highest combined score (89.14), and an RDB of 6.0, consistent with an aromatic ring plus additional unsaturation/oxidation typical of catechol-derived conjugates. Alternative formulas were less credible in this biological context: H_3_O_7_N_7_ requires an unrealistically high nitrogen/oxygen content; C_14_HON_2_ demands very high unsaturation (RDB = 15.5) and is not a plausible catechol derivative; and CH_7_O_2_N_7_S_2_ proposes two sulfurs, which would be expected to produce a more pronounced A+2 isotope contribution than observed. MS/MS data further support C_8_H_7_O_4_NS. CID and HCD spectra (Figure S4) show intense, sulfur-diagnostic fragments SO_3_^−^ at 79.9571 *m*/*z* (Δ*m*/*z* = 0.0008) and HSO_3_^−^ at m/z 80.9650 (Δ*m*/*z* = 0.0009), consistent with cleavage of the C-S bond from a cysteine-containing conjugate. The complementary ion 132.0453 *m*/*z* (C_8_H_6_ON^−^; Δ*m*/*z* = 0.0009) corresponds to the catechol-nitrogen fragment formed by neutral loss of SO_3_ from the precursor, and further fragmentation yields m/z 92.0503 (C_6_H_6_N^−^; Δ*m*/*z* = 0.0008). The precursor/product relationships and <0.001 m/z difference with theoretical masses reinforce a cysteine adduct. From a biological perspective, C_8_H_7_O_4_NS is consistent with a catechol-cysteine conjugate formed after oxidation of catechol to an o-quinone followed by thiol (cysteine) Michael addition within the mercapturic acid pathway (MAP). The presence of both nitrogen and sulfur in the formula and efficient ionization in negative mode aligns with expectations for a polar sulfur-containing intermediate that is rapidly processed to downstream N-acetylcysteine derivatives. Together, mass accuracy, a chemically reasonable RDB, corroborating MS/MS fragments and biological context support assignment of Peak 3 to C_8_H_7_O_4_NS.

**Figure 3:**
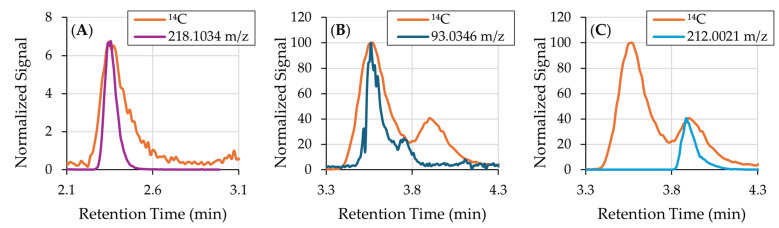
^14^C chromatogram overlaid with EICs obtained by the orbitrap mass analyzer. (**A**) Peak 1 aligned with the EIC of 218.1034 *m*/*z*; (**B**) Peak 2 aligned with the EIC of 93.0346 *m*/*z*; and (**C**) Peak 3 aligned with the EIC of 212.0021 *m*/*z*. Band broadening of the ^14^C peaks are a result of the combustion process of the moving wire interface and longitudinal diffusion in the capillaries that transfer the gaseous sample from the interface to the AMS source.

Figure 3 supports the identifications of Peaks 1–3 by demonstrating close co-elution of the ^14^C peaks with their assigned ions. Overlays of the EICs on the ^14^C chromatogram show highly similar peak shapes. Minor discrepancies—broader ^14^C features, tailing peaks and small RT shifts—are expected and attributable to band broadening introduced during combustion on the moving wire interface and to longitudinal diffusion as CO_2_ is transferred via capillaries to the AMS source. These observations are consistent with true co-elution and support the proposed identities for Peaks 1–3.

**Figure 4:**
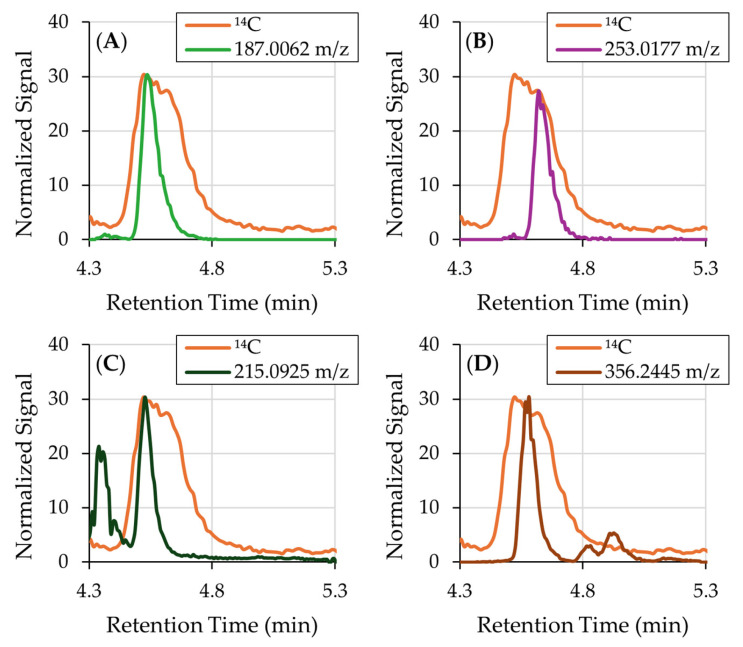
(**A**) ^14^C chromatogram overlaid with EICs obtained by the orbitrap mass analyzer demonstrating ions that coelute with Peak 4. (**A**) 187.0062 *m*/*z* and (**B**) 253.0177 *m*/*z* are not broad enough to match the peak shape. (**C**) 215.0925 *m*/*z* and (**D**) 356.2445 *m*/*z* both are not broad enough and have extraneous unlabeled peaks.

Despite extensive analysis, no single ion could be definitively assigned to Peak 4. Several ions with matching retention times were detected, including 187.0062, 215.0925, 253.0177, and 356.2445 *m*/*z* (shown in Figure 4). However, none of these ions exhibited peak shapes consistent with the ^14^C signal, suggesting that they do not contain the radiolabel and are therefore unrelated to the administered catechol. We performed targeted MS searches of known catechol metabolites, including benzoquinone, phenol, hydroquinone, and 1,2,4-benzenetriol, but no matching peaks were found. The broad and irregular shape of Peak 4 further suggests that it may represent a composite of two or more co-eluting ^14^C-labeled metabolites, rather than a single species. It is also possible that Peak 4 contains low-ionizing or thermally labile conjugates (e.g., metabolites undergoing in-source fragmentation or degradation). The complexity of the ^14^C profile and the absence of a clear molecular signal in Orbitrap data precluded identification of Peak 4. Future work will focus on the use of untargeted MS/MS, orthogonal LC methods for improved separation, and expanded ionization modes (positive ionization and atmospheric pressure chemical ionization (APCI).

Table 4 contains a summary of Peak identifications. Both Peak 1 and Peak 3 received Confidence Level 4 (Molecular Formula), as defined by Schrimpe-Rutledge et al. [39], due to the absence of definitive structural assignment. Peak 2 was attributed to benzene oxide (C_6_H_6_O), a known epoxide intermediate of catechol oxidation; although MS/MS fragmentation was not observed, the structural context and retention time supported a Confidence Level 3 (Tentative Structure) annotation. MS/MS fragments are annotated in Table S1, and a brief description of the Confidence Levels can be found in Table S2. Peak 4 remains unidentified, with no matching MS feature and a broad ^14^C signature, indicating a possible composite of multiple unresolved species.

### 3.2. Differential Metabolite Expression

**Figure 5:**
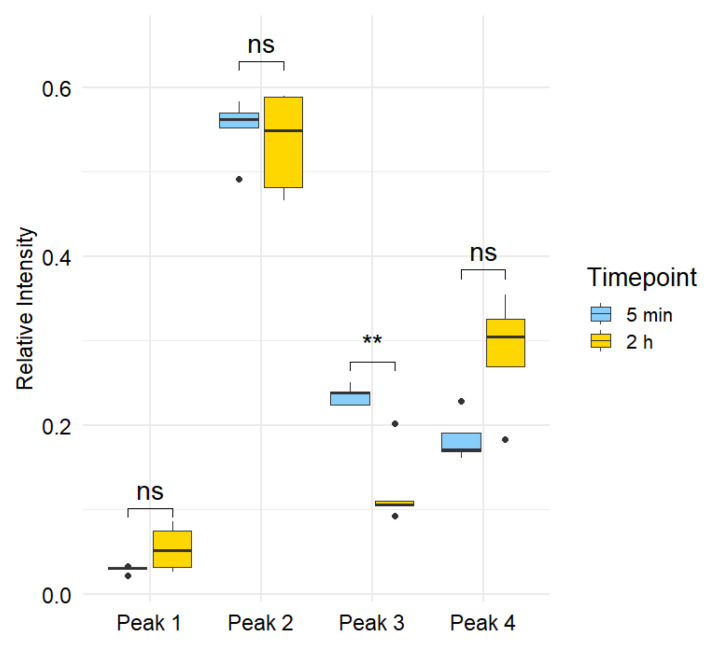
Statistical comparison of relative peak areas at 5 min vs. 2 h. For each run, the relative abundance of each peak was computed as its peak area divided by the total peak area of the four ^14^C peaks. Paired observations from the same rat (n = 5) were compared with a paired *t*-test; *p*-values were adjusted using the Benjamini-Hochberg FDR procedure. Only Peak 3 showed a significant decrease over time (raw *p* = 0.0021; FDR = 0.0086; denoted by **), whereas Peaks 1, 2, and 4 were not significant (FDR > 0.05; denoted by ns).

To assess temporal changes in metabolite levels, relative peak intensities were compared between plasma samples collected at 5 min and 2 h post-instillation. Each rat contributed matched samples at both timepoints, allowing for within-subject comparisons and increased statistical power. For each chromatographic run, the peak area was calculated for all four ^14^C-labeled peaks. To account for variability in total radiocarbon signal across samples due to decreased reproducibility of liquid-sample AMS when ^14^C signal is low [14], the relative abundance of each peak was determined by expressing its area as a fraction of the total area across all four peaks within the same run (results shown in Table S3). The relative peak areas were then averaged across biological replicates for each timepoint (5 min and 2 h). To evaluate temporal differences in metabolite distribution, the relative abundances at 5 min were statistically compared with those at 2 h using a paired t-test. Multiple comparisons were corrected using a false discovery rate (FDR) approach, ensuring robust control of type I error while maintaining sensitivity to detect meaningful changes in metabolite profiles over time. Results are shown in Table S4 and plotted in Figure 5.

Statistical comparison of relative peak areas between the 5 min and 2 h timepoints revealed that only one of the four peaks exhibited a significant temporal change. Peak 3 showed a statistically significant decrease in relative abundance over time, with a raw *p*-value of 0.0021 and an FDR-adjusted *p*-value of 0.0086, meeting the significance threshold of FDR < 0.05. In contrast, Peaks 1, 2, and 4 did not show significant changes in abundance over time, with FDR-adjusted *p*-values of 0.1244, 0.6586, and 0.1127, respectively. These results indicate that among the detected metabolites, only Peak 3, a catechol-cysteine conjugate with the formula C_8_H_7_O_4_NS, exhibits rapid turnover or clearance within the first two hours following exposure, consistent with its proposed role as a short-lived intermediate in catechol metabolism.

The pronounced, early decline of Peak 3 underscores the transient nature of the catechol-cysteine conjugate and points toward rapid engagement of the mercapturic acid pathway. In this detoxification cascade, electrophilic xenobiotics are initially conjugated with glutathione, enzymatically processed to cysteinylglycine and cysteine adducts, and finally N-acetylated to yield mercapturic acids that are efficiently excreted in urine [40]. The short residence time of the cysteine conjugate observed here is consistent with its position as an intermediate: after formation in the respiratory tract or bloodstream, it is promptly converted to downstream metabolites or cleared via renal filtration [41]. Such rapid turnover suggests that peak plasma levels of catechol MAP intermediates occur within minutes of exposure, making them fleeting biomarkers of acute smoke inhalation. Conversely, the stability of Peaks 1, 2, and 4 indicates either slower metabolic flux or incorporation into alternative biotransformation routes. These findings highlight the importance of sampling at very early time points to capture transient MAP intermediates.

### 3.3. Summary

PAMMS enabled sensitive, blood-based tracing of intranasally administered ^14^C-catechol and its circulating metabolites, resolving four radiocarbon peaks and assigning three to plausible structures: a glutamine conjugate (Peak 1), benzene oxide (Peak 2), and a cysteine conjugate (Peak 3). Parent catechol was not observed, indicating rapid biotransformation upon entry into circulation. Co-elution of ^14^C peaks with orbitrap features and consistent peak shapes support these assignments, and the significant decline of Peak 3 from 5 min to 2 h is consistent with a short-lived intermediate in the mercapturic acid pathway. These results demonstrate that PAMMS can simultaneously provide quantitative radiocarbon tracing and molecular identification from small plasma volumes—capabilities well suited to ADME/PK studies at environmentally relevant doses. Remaining uncertainties include the composite nature of Peak 4 and the need for definitive structural confirmation. Future experiments will apply targeted LC-MS/MS fragmentation, comparison to authentic standards, and complementary spectrometric techniques to validate the structures of the identified metabolites. PAMMS could also be used for smoke phenolics and other ^14^C-labeled xenobiotics to support biomarker discovery and pharmacokinetic modeling. In addition to environmental toxicology, this protocol may be adapted for preclinical drug development, microdosing studies, or chemical risk assessment scenarios that demand high sensitivity and structural insight from trace-level exposures.

## Figures and Tables

**Table 2:** Elemental formula predictions for the ion at 93.0346 *m*/*z* generated by Thermo Scientific FreeStyle software. Candidate formulas are ranked by mass accuracy (*m*/*z* delta), rings plus double bonds equivalents (RDB), and a combined score that integrates isotopic fit, heuristic rules, and elemental plausibility.

Formula	[M-H]^−^	*m*/*z* Delta	RDB	Combined Score
C_6_H_6_O	93.0335	0.0011	4.0	89.71
C_4_H_4_N_3_	93.0322	0.0024	4.5	87.60
H_7_N_4_P	93.0325	0.0021	0.0	86.29
C_2_H_9_ONP	93.0338	0.0008	−0.5	83.26

**Table 3:** Elemental formula predictions for the ion at 212.0021 *m*/*z* generated by Thermo Scientific FreeStyle software. Candidate formulas are ranked by mass accuracy (*m*/*z* delta), rings plus double bonds equivalents (RDB), and a combined score that integrates isotopic fit, heuristic rules, and elemental plausibility.

Formula	[M-H]^−^	*m*/*z* Delta	RDB	Combined Score
C_8_H_7_O_4_NS	212.00230	−0.0002	6.0	89.14
H_3_O_7_N_7_	212.00210	0.0000	3.0	76.99
C_14_HON_2_	212.00160	0.0005	15.5	76.69
CH_7_O_2_N_7_S_2_	212.00300	−0.0009	2.5	76.52

**Table 4:** Summary of ^14^C-labeled peak identifications from intranasally administered ^14^C-catechol in rat plasma. The table lists the mass-to-charge ratios (*m*/*z*), elemental formulas, proposed metabolite identities, and confidence levels based on the classification framework by Schrimpe-Rutledge et al. [39].

Peak	*m*/*z*	Formula	Proposed Identity	Confidence Level
1	218.1034	C_9_H_17_O_5_N	catechol-glutamine conjugate	4: Molecular Formula
2	93.0346	C_6_H_6_O	benzene oxide	3: Tentative Structure
3	212.0021	C_8_H_7_O_4_NS	catechol-cysteine conjugate	4: Molecular Formula
4	N/A	N/A	N/A	N/A

## Data Availability

The raw data supporting the conclusions of this article will be made available by the authors on request.

## Supplementary Materials

The following supporting information can be downloaded at: https://www.mdpi.com/article/10.3390/mps8060147/s1. Figure S1: Representative chromatograms obtained by PAMMS analysis of rat plasma after ^14^C-labeled catechol administration. Figure S2: CID and HCD mass spectra of the 218.1034 *m*/*z* precursor ion. Figure S3: CID and HCD mass spectra of the 93.0346 *m*/*z* precursor ion. Figure S4: CID and HCD mass spectra of the 212.0021 *m*/*z* precursor ion. Table S1: Annotated MS/MS fragments for Peaks 1–3. Table S2: Summary of confidence levels for metabolite identifications, as defined by Schrimpe-Rutledge et al. Table S3: Relative intensities of ^14^C peaks from plasma of five rats. Table S4: Statistical comparison of ^14^C peaks from plasma.

## Abbreviations

The following abbreviations are used in this manuscript:

AAALAC	Association for Assessment and Accreditation of Laboratory Animal Care
ADME	Absorption, Distribution, Metabolism and Excretion
AMS	Accelerator Mass Spectrometry
APCI	Atmospheric Pressure Chemical Ionization
AUC	Area Under Curve
CID	Collision-Induced Dissociation
ECD	Electron Capture Dissociation
EIC	Extracted Ion Chromatogram
EPA	Environmental Protection Agency
ETD	Electron Transfer Dissociation
FDR	False Discovery Rate
HCD	Higher-Energy Collision-Induced Dissociation
HESI	Heated Electrospray Ionization
HR	High-Resolution
IACUC	Institutional Animal Care and Use Committee
IRMPD	Infrared Multiphoton Dissociation
LC	Liquid Chromatography
LLNL	Lawrence Livermore National Laboratory
MAP	Mercapturic Acid Pathway
MS	Mass Spectrometry
NIGMS	National Institute of General Medical Sciences
NIH	National Institutes of Health
PAMMS	Parallel Accelerator and Molecular Mass Spectrometry
PK	Pharmacokinetic
PM	Particulate Matter
PM_2.5_	Particulate matter less than 2.5 μm diameter
RDB	Rings plus Double Bonds
RF	Radio Frequency
RP	Reverse-Phase
RT	Retention Time
SPE	Solid-Phase Extraction
TIC	Total Ion Chromatogram
UVPD	Ultraviolet Photodissociation
WSE	Wood Smoke Extract
