# Supplementary material for: Identification and Quantitation of 14C-Labeled Catechol Metabolites in Rat Plasma After Intranasal Instillation of Smoldering Eucalyptus Wood Smoke Extract
Source: Methods Protoc. 2025 Dec 4;8(6):147. doi: 10.3390/mps8060147 (PMC12735648; doi:10.3390/mps8060147)
Supplement: Supplementary file 1 [file mps-08-00147-s001.zip › mps-3902572-supplementary.pdf]

methods and protocols

MDPI

Supplementary Information for

Identification and Quantitation of ^{14}C-Labeled Catechol Metabolites in Rat Plasma after Intranasal Instillation of Smoldering Eucalyptus Wood Smoke Extract

David Baliu-Rodriguez [1*], Dorothy J. You [1], Michael Malfatti [1], Esther Ubick [1], Yong Ho Kim [2] and Bruce A. Buchholz [3*]

[1] Biosciences and Biotechnology Division, Lawrence Livermore National Laboratory, Livermore, CA 94550, USA

[2] Risk Assessment Support Division, Office of Mission Critical Operations, Office of Chemical Safety and Pollution Prevention, U.S. Environmental Protection Agency, Research Triangle Park, NC 27711, USA

[3] Center for Accelerator Mass Spectrometry, Lawrence Livermore National Laboratory, Livermore, CA 94550, USA

* Correspondence: Bruce A. Buchholz, buchholz2@llnl.gov; David Baliu-Rodriguez, baliurodrigu1@llnl.gov

Table of Contents

Figure S1 .... **S3**

Figure S2 .... **S4**

Figure S3 .... **S5**

Figure S4 .... **S6**

Table S1 .... **S7**

Table S2 .... **S8**

Table S3 .... **S9**

Table S4 .... **S10**

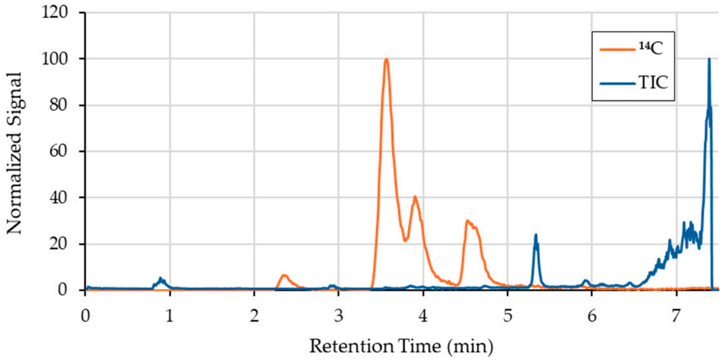

Figure S1. A representative result obtained by PAMMS analysis of rat plasma after ^{14}C-labeled catechol administration. Fullscan MS with an Orbitrap Eclipse was used to obtain the total ion chromatogram (TIC) and overlaid with the ^{14}C chromatogram collected by AMS. All ten analyzed samples had a consistent chromatographic profile.

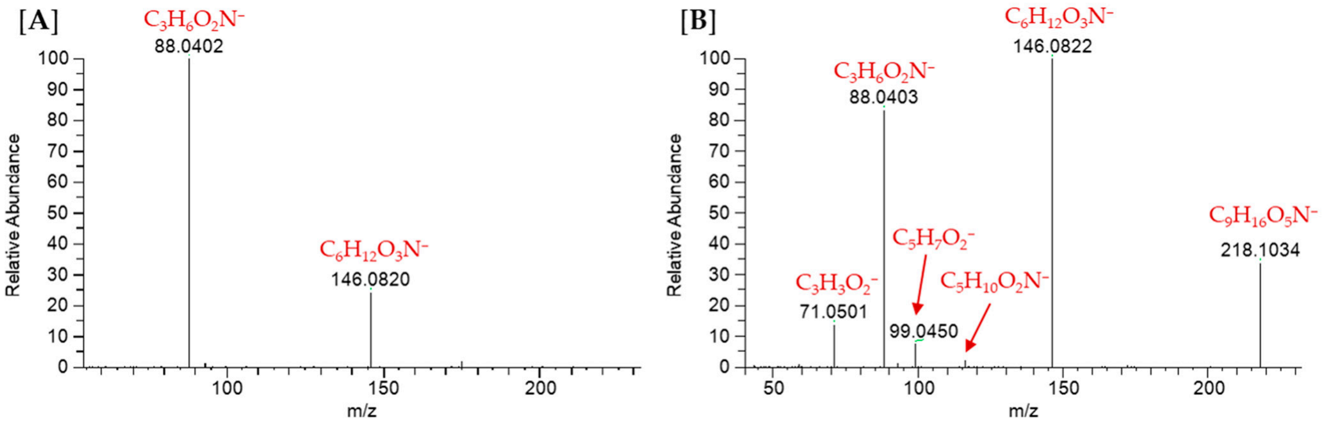

Figure S2. [**A**] CID and [**B**] HCD mass spectra of the 218.1034 m/z precursor ion with labeled fragment ions.

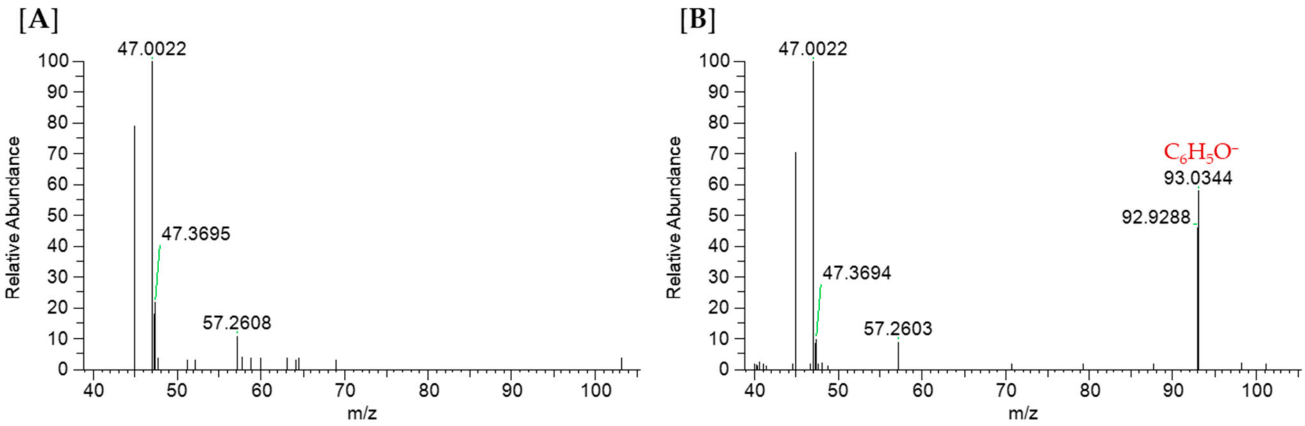

Figure S3. [**A**] CID and [**B**] HCD mass spectra of the 93.0346 m/z precursor ion. The displayed fragment ions were contaminants present throughout the chromatogram.

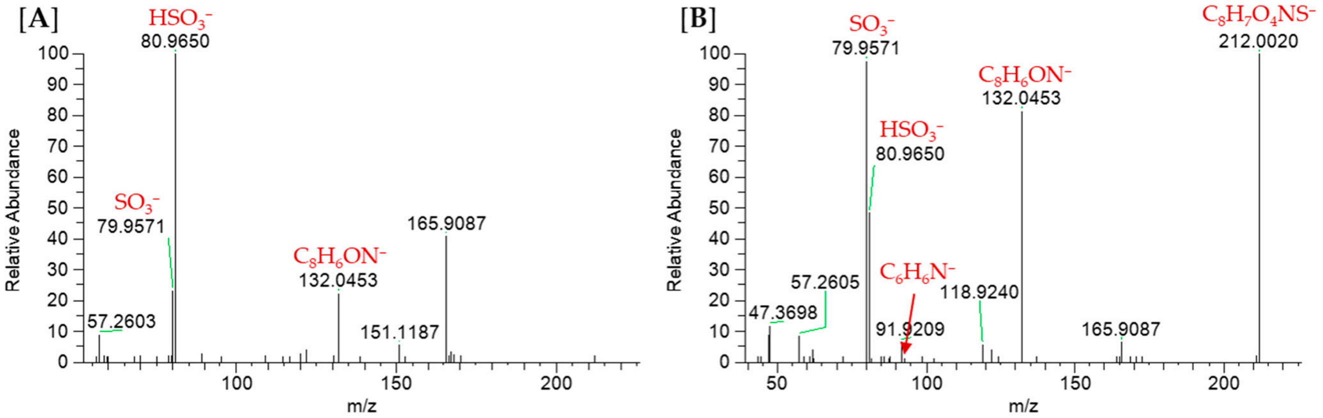

Figure S4. [**A**] CID and [**B**] HCD mass spectra of the 212.0021 m/z precursor ion with labeled fragment ions.

Table S1. Annotated MS/MS fragments for Peaks 1-3. Peak 1 was tentatively identified as a catechol-glutamine conjugate, Peak 2 as benzene oxide, and Peak 3 as a catechol-cysteine conjugate.

Peak	m/z	Supporting MS/MS Fragments
1	218.1034	$C_6H_{12}O_3N^-$, $C_5H_{10}O_2N^-$, $C_3H_6O_2N^-$, $C_5H_7O_2^-$, $C_3H_3O_2^-$
2	93.0346	None
3	212.0021	$C_8H_6ON^-$, $C_6H_6N^-$, SO_3^-, HSO_3^-

Table S2. Summary of confidence levels for metabolite identifications, as defined by Schrimpe-Rutledge et al.

Confidence Level	Description
Level 5	Unique Feature: mass measurement accuracy
Level 4	Molecular Formula: isotope abundance distribution, charge state and adduct ion determination
Level 3	Tentative Structure: MS1 m/z database match
Level 2	Putative Identification: MS/MS spectrum match
Level 1	Validated Identification: reference standard confirms structure

Table S3. Relative intensities of ^{14}C peaks from plasma of five rats. For each chromatographic run, the peak area was calculated for all four ^{14}C-labeled peaks, and the relative abundance of each peak was determined by expressing its area as a fraction of the total area across all four peaks within the same run.

Rat	Timepoint	Peak 1	Peak 2	Peak 3	Peak 4
1	5 min	0.033	0.552	0.224	0.191
	2 h	0.075	0.466	0.105	0.355
2	5 min	0.030	0.57	0.239	0.161
	2 h	0.086	0.482	0.106	0.326
3	5 min	0.022	0.584	0.223	0.171
	2 h	0.052	0.549	0.093	0.305
4	5 min	0.030	0.492	0.251	0.228
	2 h	0.026	0.589	0.202	0.183
5	5 min	0.030	0.563	0.238	0.169
	2 h	0.032	0.590	0.110	0.269

Table S4. Relative intensities of ^{14}C peaks were statistically compared at 5 min with 2 h using a paired t-test. Multiple comparisons were corrected using a false discovery rate (FDR) approach.

Peak	5 min average	2 h average	p-value	FDR-adjusted p-value
1	0.029	0.053	0.0933	0.124
2	0.552	0.534	0.659	0.659
3	0.235	0.127	0.0021	0.0086
4	0.184	0.286	0.0563	0.113

Support was provided by the LLNL-LDRD Program under Projects 23-ERD-020 and 24-LW-026, and by NIH R24GM137748. This work was performed in part at Lawrence Livermore National Laboratory under the auspices of the U.S. Department of Energy under contract DE-AC52-07NA27344. Reviewed and released as LLNL-JRNL-2011012.
